# Antibiotic resistance pattern of *Pseudomonas aeruginosa* isolated from urine samples of Urinary Tract Infections patients in Karachi, Pakistan

**DOI:** 10.12669/pjms.312.6839

**Published:** 2015

**Authors:** Dania Aijaz Shah, Shehnaz Wasim, Farhan Essa Abdullah

**Affiliations:** 1 Dania Aijaz Shah, Dow University of Health Sciences, Baba-E-Urdu Road, Karachi 74200, Pakistan; 2 Shehnaz Wasim, Ziauddin University, 4/B, Shahrah-e-Ghalib, Block 6, Clifton, Karachi - 75600, Pakistan; 3 Farhan Essa Abdullah, Dr. Essa Laboratory and Diagnostic Centre, B-122, Block-H, Shahrah-e-Jahangir, North Nazimabad, Karachi, Pakistan

**Keywords:** *Pseudomonas aeruginosa*, Urinary tract infections, Antibiotic resistance pattern

## Abstract

**Objective:**

The aim of this study was to evaluate the antibiotic resistance pattern of *Psedomonas aeruginosa* and its prevalence in patients with urinary tract infections (UTI) for effective treatment in a developing country like Pakistan.

**Methods:**

This is an observational study conducted for a period of ten months which ended on December 2013 at the Dr. Essa Laboratory and Diagnostic Centre in Karachi. A total of 4668 urine samples of UTI patients were collected and standard microbiological techniques were performed to identify the organisms in urine cultures. Antibiotic susceptibility testing was performed by Kirby-Bauer technique for twenty five commonly used antimicrobials and then analyzed on SPSS version 17.

**Results:**

*P. aeruginosa* was isolated in 254 cultures (5.4%). The most resistant drugs included Ceclor(100%) and Cefizox (100%) followed by Amoxil/Ampicillin (99.6%), Ceflixime (99.6%), Doxycycline (99.6%), Cefuroxime (99.2%), Cephradine (99.2%), Cotrimoxazole (99.2%), Nalidixic acid (98.8%), Pipemidic acid (98.6%) and Augmentin (97.6%).

**Conclusion:**

Emerging resistant strains of *Pseudomonas aeruginosa* are potentially linked to injudicious use of drugs leading to ineffective empirical therapy and in turn, appearance of even more resistant strains of the bacterium. Therefore, we recommend culture and sensitivity testing to determine the presence of *P.aeruginosa* prior to specific antimicrobial therapy.

## INTRODUCTION

Urinary Tract Infection (UTI) is one of the leading causes of infection worldwide. Although *E-coli* is predominantly associated with the etiology of UTI,^1^ other organisms such as *Klebsiella pneumonia*, *Proteus mirabilis*, *Staphylococcus aureus* and most importantly *P.aeruginosa* are on the rise.^2^

Multi-drug resistant (MDR) bacteria have been defined as ‘resistant to one agent in three or more antibiotic categories’.^3^ Unfortunately, *P.aeruginosa* has proven to be one of them in our study, thus making its treatment ineffective. It is a gram-negative rod that can survive in myriad of environments such as aquatic and terrestrial.^4^ It contributes strongly to nosocomial infections and affects immunocompromised patients.^4^ The inherent antimicrobial resistance mechanisms of *P. aeruginosa* which include lower outer membrane permeability, increased expression of efflux pumps of different specificity and presence of Amp C beta-lactamase wreak havoc by granting the bacteria resistance mechanisms to commonly used antibiotics namely Penicillin G, first and second generation of cephalosporins and quinolones.^5^

We are confronted with limited therapeutic options due to the acquisition of resistance mechanisms by these pathogens chiefly because of the indiscrete use of antibiotics, failure to complete therapeutic regimens and variation in the doses administered along with over the counter availability.^6^ This may explain the fact that about 150 million cases of UTI are witnessed annually, making it the third most diagnosed infection.^7^,^8^ This collectively results in higher rates of morbidity and mortality, and accounts for the immense cost incurred on our healthcare system. Therefore, there is an increased need for newer antibiotics to treat UTI efficaciously.

Our study focuses on the prevalence and antibiotic resistance pattern of *P.aeruginosa*. We have assessed and compared our findings with the national and international antimicrobial patterns and have investigated drugs for the effective treatment of *P.aeruginosa*. We also briefly draw attention to the relation of antimicrobial patterns with the intrinsic and acquired antimicrobial mechanisms of *P.aeruginosa*.

## METHODS

### Research subject and design

The study includes both nosocomial and community- acquired isolates of *P.aeruginosa*. A total of 4668 UTI cases were reviewed in this study from March 2013 to December 2013. Midstream specimen of urine, preferably of first morning void was collected of known UTI patients. The samples were then taken to the lab within two hours and standard microbiological procedures were performed to detect the presence of *P.aeruginosa* species.

### Antimicrobial resistance testing

Antimicrobial resistance testing was performed and analyzed by Kirby Bauer technique on Mueller-Hinton agar plates using twenty five antibiotics according to their respective break points. NCCLS (1987) and WHO guidelines were followed to determine the zone diameters consistent with the Zone Size Interpretative Chart. Data was analyzed using SPSS version 17.

## RESULTS

*E-coli* was the predominant organism (42.9%). Out of the total 4668 samples, *P.aeruginosa* grew in 254 cultures (5.4%). Other organisms detected were *Klebsiella spp* (18.4%), *Staphylococcus spp* (18.1%), *Enterococci* (4.8%), *Enterobacter* (3.5%), *Salmonella spp* (0.77%) and *Proteus spp* (0.64%). *Candida* was found in 5.4% of the cases.

**Fig.1:**
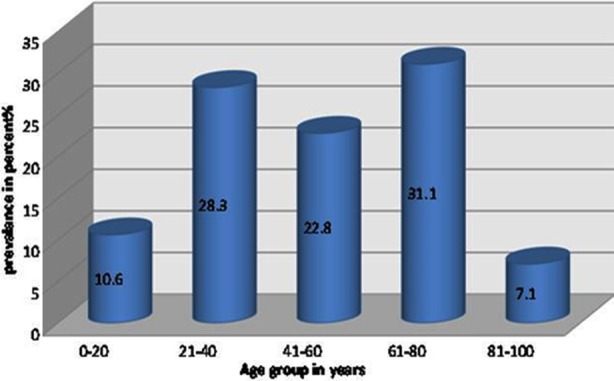
Prevalence of Pseudomonas aeruginosa in different age groups.

Amongst the 254 *P.aeruginosa* infected UTI patients, 64.71% were females and 35.29% were males. Highest number of cases were seen in the age group 61-80 followed by the age group 21-40 while patients ranging from 81 to 100 years were least affected (Fig.1).

Interestingly, certain cases showed the presence of other organisms along with *P.aeruginosa*. *Enterococcus spp* was found in 11 cases (4.3%), *Staphylococcus spp* in 6 (2.4%), *E-coli* in 3 (1.2%) and *Candida* in 11 (4.3%). Table-I displays the overall patterns of antimicrobial resistance of *P.aeruginosa*. Analysis of the disk-diffusion tests in vitro revealed that *P.aeruginosa* was sensitive to Imipenem, Piperacillin/Tazobactam and Amikacin. Resistance was observed to Ceclor, Cefizox, Amoxil/Ampicillin, Ceflxime, Doxycycline, Cefuroxime, Cephradine, Cotrimoxazole, Nalidixic acid, Pipemidic acid and Augmentin.

**Table-I:** Resistivity pattern of *P.aeruginosa* towards various antibiotics. (n = 254).

Resistance	Drugs
(%)	Number(percentage)				
100	Ceclor
100	Cefizox
99.6	Amoxil/Ampicillin
99.6	Ceflxime
99.6	Doxycycline
99.2	Cefuroxime
99.2	Cephradine
99.2	Cotrimoxazole
98.8	Nalidixic acid
98.6	Pipemidic acid
97.6	Augmentin
63.9	Cefepime
63.9	Fosfomycin
61.7	Ceftriaxone
58.4	Tobramycin
56.1	Ceftazidime
50.4	Enoxacin
50.2	Sparfloxacin
50	Ciprofloxacin
49	Ofloxacin
45.5	Cefotaxime
35.3	Gentamicin
25.3	Amikacin
19.6	Piperacillin/Tazobactam
10.4	Imipenem

## DISCUSSION

Majority of the *P.aeruginosa* related UTI cases occurred in females as compared to males. Chances of contracting UTI increase by 1% per decade for women.^8^ In our study, patients in the age group of 61-80 were most affected. This could be explained by an increase in the incidence of prostatic hypertrophy in men after the age of 55. Women, on the other hand, are prone to conditions such as vaginitis, atrophy and prolapse of womb or vagina which set up the path for UTI.^8^,^9^

The age group 21-40 was second most affected possibly due to the increase in sexual activity, higher number of pregnancies and use of certain types of contraception that can cause UTI for example diaphragm or spermicide.^8^ It is reported that persistent forms of UTI affect less than 2% of the school-going girls.^8^

In our study, percentage of *P.aeruginosa* was 5.4% which was close to the percentage reported in a European study as 6.9%.^10^ In Kashmir, *P.aeruginosa* accounted for 7.6% of the urine samples^11^ while in Nepal, *P.aeruginosa* encompassed 1.20% of the total cultures.^12^ Study from India showed prevalence of *P.aeruginosa* to be 9.3%^7^ while 9% of the bacterial dominance was found in Palestine.^13^ Retrospective studies in Pakistan show prevalence of the bacteria to be 9.2% and 3.27%.^16^,^17^ According to our study, *P.aeruginosa* in UTI patients can be best treated with Imipenem with minimum resistance (10.4%), followed by Piperacillin/Tazobactam (19.6%) and then Amikacin (25.3%). A similar study by Naeem et al from Pakistan showed 99-100% effectiveness of Amikacin and improved therapeutic outcomes with Imipenem and Piperacillin/Tazobactam against *P.aeruginosa*. 14 However a study from India reported resistance of *P.aeruginosa* to Amikacin which was in contradiction to our study and that of Naeem et al.^14^,^15^ Among other aminoglycosides, Gentamicin’s level of resistance was 35.3% against *P.aeruginosa* while two studies from Pakistan showed this antibiotic’s efficacy to be 69.2% and 83%.^16^,^17^ In a European study, nosocomial isolates of *P.aeruginosa* showed 72% resistivity towards Gentamicin, 69.2% to Tobramycin and 40% to Amikacin.^10^

This could be associated with the growing resistance of gram-negative bacteria including *P.aeruginosa* to Gentamicin. Amikacin has been used sparingly only in severe forms of the disease owing to high treatment costs and the intravenous nature of administration. Therefore, drug resistance has been slow to emerge.

*P.aeruginosa* resists antimicrobial drugs either through acquiring beta-lactamases especially extended-spectrum enzymes, carbapenemases or aminoglycoside-modifying enzymes.^18^ Resistance is conferred by the transfer of plasmids which carry genes to produce antimicrobial enzymes. Plasmids are passed on to the bacterial progeny by replication and to other members by conjugation, generating a bacterium populace that becomes harder to treat with the passage of time.^19^,^20^

Antibiotic resistance correlates well with the frequency of drug use and in a state like Pakistan where drugs are easily available over-the-counter, bacterial resistance to antibiotics grows rapidly, putting our health care system in a conundrum. Drug resistance facilitates growth and increases prevalence of persistent pathogens which become difficult to exterminate. Studies previously have shown the emergence of resistant genes in bacteria within 5 years of antibiotic intervention.^21^

Imipenem (carbapenem) shows remarkable activity in our study and this could be attributed to its infrequent use in the treatment of UTI patients, however, Carbapenem-hydrolyzing strains of *P.aeruginosa* have been isolated in clinical settings of some developed countries such as Italy, China, Japan and Singapore.^5^ Carbapenem-hydrolyzing strains of *P.aeruginosa* resist carbapenems (Imipenem and Meropenem).^5^ The only monobactam possibly effective against carbapenem-hydrolyzing strains of *P.aeruginosa* is Aztreonam.^5^ Frequent use of Imipenem changes the outer membrane permeability or in seldom cases when given in collaboration with Piperacillin, modifies the target sites of *P.aeruginosa* which then becomes resistant to carbapenems.^5^

Growing resistance of Ciprofloxacin and Cefotaxime was found in *P.aeruginosa* in previous studies which was quite similar to the resistivity pattern we found of Cefotaxime (45.5%) and Ciprofloxacin (50%).^15^

In our study, Ofloxacin showed the greatest level of activity in quinolones against the resistant bacterium being 51% sensitive, followed by Ciprofloxacin which was 50% sensitive, Sparfloxacin (49.8%) and Enoxacin (49.6%). However, another study from Pakistan showed resistance pattern of Ciprofloxacin to be 25%.^22^ The same study showed the resistance of Nalidixic acid and Pipemidic acid to be 87% and 25% respectively.^22^ However in our study the resistance of these two drugs was found to be 98.8% and 98.6% respectively, validating the stifled therapeutic action of the quinolones due to frequent use.^22^ A study from Nigeria found the resistance levels of Ciprofloxacin, Sparfloxacin and Ofloxacin to be 77.1%, 44.8% and 19.8% respectively.^23^

Quinolones are preferred empiric therapy for UTI before the physicians receive culture results mainly because of the easy availability in oral forms and reasonable cost.^24^ However, emerging quinolone resistant strains of *P.aeruginosa* have surfaced owing to the changes in target enzymes of the bacteria and active efflux pumps generated to prevent the entry of the drugs.^5^

Fosfomycin was considered a sensitive drug at 66% against *P.aeruginosa* in another study from Pakistan while the level of resistance in our study was 63.9%.^17^ Among the cephalosporins, Cefotaxime was most effective against *P.aeruginosa* with only 45.5% resistivity level, followed by Ceftazidime (56.1%) and Cefepime (63.9%). Ceftriaxone’s resistance level was 80% in a study from India while in our study it was 61.7%.^2^

A study from Palestine, showed resistivity level of Ceftazidime to be 13.3% and that of Cefuroxime to be 53.3% while in our study it was 56.1% and 99.2%, respectively.^13^ Although susceptibility levels of Ceftazidime and Ceftriaxone found by Paryani et al which were 97% and 87% respectively, differs greatly with that found in our study, it does prove the relative efficacy of these drugs amongst the cephalosporins against *P.aeruginosa*. 17 The resistance rate of Cotrimoxazole is 99.2% in our study which was similar to that found by Manjunath GN et al. (100%).^7^

Production of beta-lactamase by *P.aeruginosa* cleaves the amide bond on the beta-lactam ring, rendering beta-lactams ineffective.^5^ Production of Amp C-beta lactamase (cephalosporinase) enables *P.aeruginosa* to resist third generation cephalosporins and surprisingly, the amount of cephalosporinase produced rises 100 to 1000 times in the presence of carbapenems like Imipenem.^5^

Thus, the only carbapenem (Imipenem) tested in our study showed the greatest level of efficacy although effectiveness of Meropenem has been noted in other studies.^25^ Aminoglycosides were the next best treatment in *Pseudomonas aeruginosa* associated UTI, followed by quinolones while cephalosporins did not fare well and similar comparisons have been reported by Paryani et al.^17^ However 4th generation cephalosporins were not tested in our study, which do show a good salutary response against some strains of *P.aeruginosa* as noted in different studies.^5^ Lastly, penicillins were highly ineffectual against *P.aeruginosa* except for Piperacillin/Tazobactam possibly because of the beta-lactamase inhibitor in addition to the extended-spectrum nature and scarce use of the drug.

While growing resistance of bacteria itself puts the future of our health care in uncertainty, what poses a greater threat is the emergence of multi-drug resistant (MDR) pathogens that would render most antibiotics ineffective by obtaining newer and complex resistance mechanisms. Some factors contributing to the rise of MDR bacteria are immunosuppression, extensive use of wide spectrum drugs and prolonged hospitalization.^25^

The antibiotic susceptibility trends investigated narrow down the most sensitive drugs from the most resistant ones and our study can be used to provide a more sound approach in improving the antibiotics therapy against the MDR *P.aeruginosa* in UTI patients.

## CONCLUSION

Ability of *P. aeruginosa* to resist antibiotics by their inherent or acquired antimicrobial mechanisms can be linked to the common practice of physicians to prescribe antimicrobials that are easily available over the counter. This trend is in conformity to global trends especially where there is no check on the purchase of drugs, confirming the rise of resistant strains of *P.aeruginosa*. Therefore we recommend careful empirical prescription, culture and sensitivity testing and selection of drugs accordingly.
